# Holzapfeliella saturejae sp. nov. isolated from flowers of winter savoury Satureja montana L.

**DOI:** 10.1099/ijsem.0.006654

**Published:** 2025-01-22

**Authors:** Cristina Alcántara, Ángela Peirotén, Luís Andrés Ramón-Núñez, José María Landete, Vicente Monedero, Manuel Zúñiga

**Affiliations:** 1 Laboratorio de Bacterias Lácticas y Probióticos, Instituto de Agroquímica y Tecnología de Alimentos (IATA-CSIC), Av. Agustín Escardino 7, 46980 Paterna, Spain; 2 Departamento de Tecnología de Alimentos, National Institute for Agricultural and Food Research and Technology (INIA-CSIC), Carretera de La Coruña Km 7.5, 28040 Madrid, Spain; 3 Valencian Institute for Agricultural Research (IVIA), 46113 Valencia, Spain

**Keywords:** flower, *Holzapfeliella*, *Satureja montana*, Valencia

## Abstract

A novel strain of the genus *Holzapfeliella*, named He02^T^, was isolated from flowers of *Satureja montana* L. in a survey for lactic acid bacteria associated with wild and cultivated plants in the metropolitan area of Valencia, Spain. Partial 16S rRNA gene sequencing revealed a similarity of 99% to *Holzapfeliella floricola* DSM 23037^T^=Ryu1-2^T^. Strain He02^T^ cells are Gram-stain-positive, catalase-negative non-motile rods, usually occurring in pairs. Cells show a pale yellow pigmentation when pelleted. As *H. floricola*, strain He02^T^ utilized a narrow range of carbohydrates, namely, glucose and fructose, homofermentatively. However, genome sequencing and estimation of average nucleotide identity (ANI) revealed an ANI value of 87.44 with *H. floricola* DSM 23037^T^, the only *H. floricola* strain sequenced to date. A value of 30.5% for digital DNA–DNA hybridization was estimated with the Type Strain Genome Server tool when He02^T^ was compared with strain DSM 23037^T^. These results indicate that strain He02^T^ constitutes a novel species, for which the name *Holzapfeliella saturejae* sp. nov. with He02^T^ (=CECT 31001^T^=DSM 117324^T^=CCM 9395^T^) as type strain is proposed.

## Introduction

Plants host diverse communities of micro-organisms, which are associated with their different organs such as roots, leaves or flowers [1]. These communities encompass a wide variety of micro-organisms and may play a beneficial role in improving growth and protection from potential pathogens. Lactic acid bacteria (LAB) are a group of low G-C, Gram-stain-positive bacteria classified within the order *Lactobacillales* which are characterized by the production of lactic acid from the fermentative degradation of carbohydrates. The order includes the families *Aerococcaceae*, *Carnobacteriaceae*, *Enterococcaceae*, *Lactobacillaceae* and *Streptococcaceae*. LAB are important starter, commensal or pathogenic micro-organisms. LAB roles in the production of fermented food products, as pathogens or as probiotics, have received the most attention. Notwithstanding, recent studies have started to identify a growing number of plant-associated LAB species and reveal their relevance as components of these microbial communities [2]. For example, fructophilic LAB have been found typically present in niches rich in fructose, such as fruits and flowers, as well as the gut of pollinating insects [3]. This group is characterized by their heterofermentative metabolism, poor growth on glucose and good growth on fructose. Interestingly, fructophilic LAB encompass LAB species belonging to different genera that have sustained genomic convergent evolution [4].

In the course of a study of the population of fructophilic LAB associated with flowering plants in the metropolitan area of Valencia, Spain, diverse LAB species were isolated. Among them, strain He02^T^ could be classified in the *Lactobacillaceae* genus *Holzapfeliella* but displayed distinctive characteristics that set it apart from *Holzapfeliella floricola*, the only species of the genus described to date. *H. floricola* was originally named *Lactobacillus floricola* [5] and later transferred to the genus *Holzapfelia* after a major revision of the classification of species of families *Lactobacillaceae* and *Leuconostocaceae* [6]. Subsequently, the genus name was changed to *Holzapfeliella* to avoid homonymy with an extinct genus of molluscs [7]. *H. floricola* strains were isolated from flowers collected in mountainous areas in Japan [5]. The type strain *H. floricola* DSM 23037^T^ was isolated from a flower of *Caltha palustris* in the Oze National Park [5]. Phenotypic characterization of *H. floricola* showed that this species degrades d-glucose to l-lactic acid homofermentatively, thus indicating that *H. floricola* is not a fructophilic LAB although it shares the same niches. This species only utilized glucose or fructose as carbon sources. Since the original description of the species [5], no further research has been carried out. Therefore, limited information is available about this genus.

## Strain isolation

Strain He02^T^ was isolated from aseptically collected flowers of *Satureja montana* L. (*Lamiaceae*; winter savoury) grown in a domestic cultivar in Lliria (Valencia, Spain; GPS coordinates 39.675, –0.613). Five flowers were transferred to a screw-capped polypropylene tube containing 5 ml of modified fructose yeast peptone broth [FYP; composition per litre: 10 g d-fructose, 10 g yeast extract, 2.5 g tryptone, 2.5 g meat extract, 2 g sodium acetate, 0.5 g Tween 80, 0.2 g MgSO_4_·7H_2_O, 0.01 g MnSO_4_·4H_2_O, 0.01 g FeSO_4_·7H_2_O, 0.01 g NaCl, 0.05 g cycloheximide and 0.05 g sodium azide (pH 6.8)] [8]. The tube was vortexed vigorously and incubated at 30 °C until turbidity was observed. Aliquots were conveniently diluted in saline solution [NaCl 0.9% (w/v)] and spread onto FYP agar plates supplemented with 5 g l^−1^ of CaCO_3_. The plates were incubated at 30 °C, and isolated colonies were picked on the basis of morphological characteristics and the presence of clear halos due to acid production. The selected colonies were streaked on FYP agar plates to ensure culture homogeneity. Gram-stain-positive and catalase-negative isolates were selected, and a single colony was inoculated in FYP broth and incubated at 30 °C for 24 h. Grown cultures were supplemented with glycerol (15% v/v final concentration) and stored at −80 °C.

**Fig. 1.**
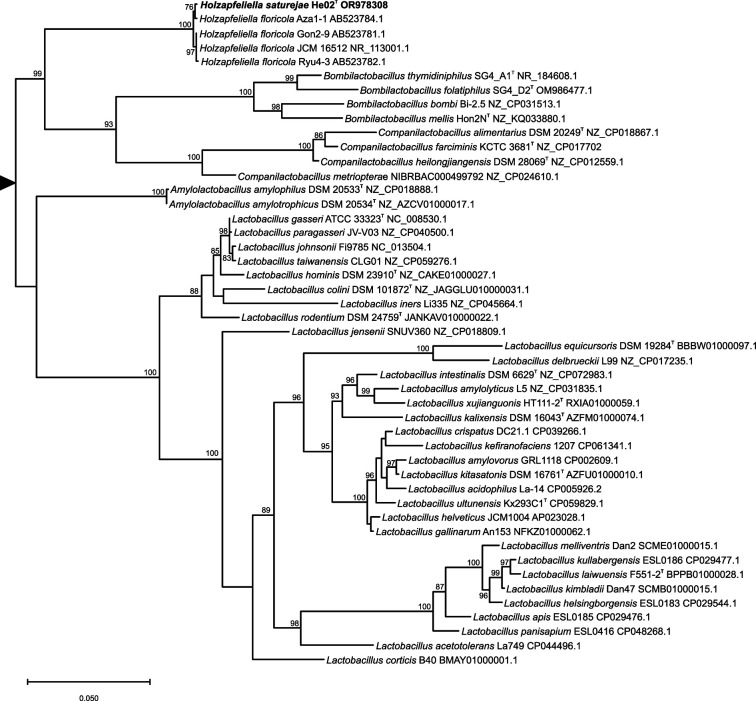
Maximum-likelihood phylogenetic tree of 16S rRNA sequences of representative *Holzapfeliella* and closely related genera strains. Support values are given for those nodes with support higher than 75%. The tree has been arbitrarily rooted for ease of visualization.

Preliminary identification of isolates was carried out by PCR amplification and partial sequencing of 16S rRNA gene with primers 27F (AGAGTTTGATCCTGGCTCAG) and 1492R (GGTTACCTTGTTACGACTT) [9]. The nucleotide blast tool (blastn) implemented in the National Center for Biotechnology Information (NCBI) web server (https://blast.ncbi.nlm.nih.gov/Blast.cgi) was used to compare the He02^T^ isolate 16S rRNA gene sequence (GenBank Acc. No. OR978308) with sequences available in the GenBank databases. Based on the blast result, He02^T^ was provisionally assigned to the genus *Holzapfeliella*. The full-length 16S rRNA sequence of He02^T^ was subsequently extracted from the whole-genome sequence (see below) and used to obtain a 16S rRNA-based phylogeny by maximum likelihood (Fig. 1). Full-length 16S rRNA sequences of *Holzapfeliella floricola* (some of them deposited as *Lactobacillus floricola*) and representative species of the most closely related genera, according to the phylogenetic reconstruction of family *Lactobacillaceae* performed by Zheng *et al.* [6], were selected. For this purpose, blastn searches directed against the selected genera were carried out using He02^T^ 16S RNA gene sequence as a query. The hits obtained were manually inspected and representative complete 16S RNA gene sequences were selected. All sequences were extracted from GenBank and aligned using muscle as implemented in mega 11.0.3 [10]. The best substitution model was selected by maximum likelihood in mega. The maximum-likelihood tree was reconstructed with IQ-TREE with the GTR+F+G4 substitution model [11]. Support values were obtained by the ultrafast bootstrap approximation [12]. The phylogenetic analysis confirmed that strain He02^T^ belongs to the genus *Holzapfeliella*. Two clusters within *Holzapfeliella* were differentiated, one containing He02^T^ and *H. floricola* Aza1-1, and the other, strains Gon2-9, JCM16512 and Ryu4-3 (Fig. 1). A recent survey of the distribution of *Lactobacillaceae* in different foods and food environments by using metataxonomic analysis based on the 16S rRNA gene also pointed, through the examination of amplicon sequence variants of the 16S rRNA V3–V4 region, to the likely existence of different and previously undescribed species within this genus [13]. This study identified *Holzapfeliella* 16S rDNA amplicons in samples derived from bovine muscle, cattle milk and cheese. Given that the only known source of *H. floricola* isolates was flowers, the presence of *Holzapfeliella* amplicons in those samples was hypothesized to be associated with pasture [13].

## Genome features of strain He02^T^

He02^T^ total DNA was extracted using an in-house cetyltrimethylammonium bromide-based purification method (to be published elsewhere) and quantified using a Qubit V3 fluorometer (Invitrogen Life Technologies). Sequencing libraries were prepared using Ligation Sequencing Kit V14 (SQK-LSK114, Oxford Nanopore) with barcoding SQK-NBD114.24. Libraries generated were sequenced in a MinION equipment using a FLO-MIN114 Vr10.4.1 flow cell (Oxford Nanopore) and duplex base-calling in the super-accuracy mode selected. The mean read size was 1867 bp with a mean read quality of 20.5 as assessed with NanoPlot v1.36.2 [14]. Reads were assembled with Flye v2.9.1 [15]. The assembly was deposited in DDBJ/ENA/GenBank (BioProject PRJNA1031542) and annotated by the NCBI Prokaryotic Genome Annotation Pipeline [16].

Recommendations outlined by Riesco and Trujillo [17] for the use of genome data for the taxonomy of prokaryotes were attended to evaluate our genomic data. A total of 1 468 086 bp were sequenced and assembled in six contigs (contig N50=1.2 Mb; contig L50=1; genome coverage 86×) with a G+C content of 34.99 mol%. The completeness and contaminations were assessed with CheckM [18] and reported as 96.24 and 0.98%, respectively. Completeness was also estimated with BUSCO [19] as implemented in the Galaxy platform [20]. A completeness of 98.51% was estimated. These values are within the thresholds (≥95% complete with ≤5% contamination) considered acceptable for taxonomy assignments [18]. The genome of *H. floricola* Ryu1-2^T^=DSM 23037^T^ (genome assembly GCA_001436605.1) is the only genome sequence available for this species and comprises 1 363 353 bp with a G+C content of 34.5 mol% . The analysis of the He02^T^ sequence identified 1403 protein-encoding genes, 13 pseudogenes and 70 RNA-encoding genes, which included 4 rRNA operons, 54 tRNAs and 3 non-coding RNAs.

**Fig. 2.**
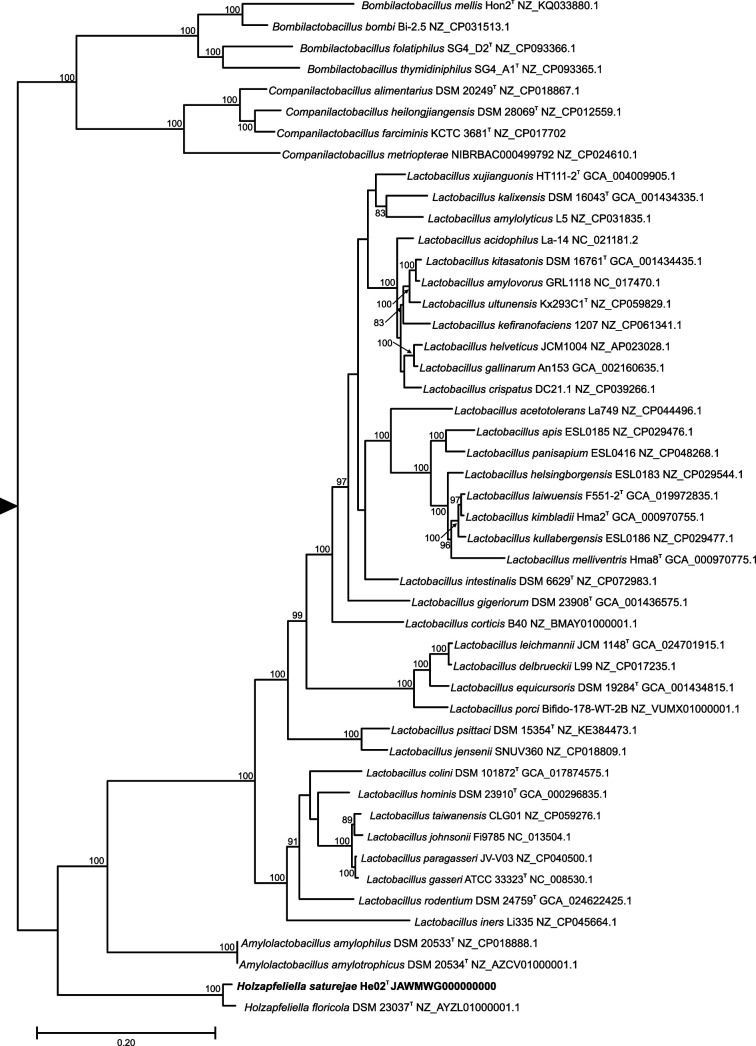
Maximum-likelihood phylogenetic tree of core genes of *Holzapfeliella* and species of closely related genera. Support values are given for those nodes with support higher than 75%. The tree has been arbitrarily rooted for ease of visualization.

In order to determine more accurately the taxonomic position of strain He02^T^, a core genome phylogeny was obtained (Fig. 2). Reconstruction of the phylogeny was derived by concatenating 20 validated core genes. The core gene set was selected to obtain the highest bacterial phylogenetic fidelity while minimizing missing data [21]. This set encompasses RNA polymerase subunits alpha and beta (*rpoA* and *rpoB*); 50S ribosomal proteins L1, L2, L3 and L5; 30S ribosomal proteins S2, S3, S5 and S7; ribosome-associated GTPases Der and CgtA; translation initiation factor IF-2; translation elongation factor 4; peptide chain release factor 1; transcription termination factor NusA; phenylalanine-tRNA ligase subunit alpha; signal recognition particle Ffh; preprotein translocase subunit SecY; and redox-regulated ATPase YchF. Protein sequences were extracted, aligned and concatenated by using VBCG v1.3 [21]. The best substitution model was selected by maximum likelihood in mega 11.0.3. The maximum-likelihood tree was reconstructed with IQ-TREE with the LG+G4+I+F+R substitution model [22]. Support values were obtained by the ultrafast bootstrap approximation implemented in IQ-TREE [12].

As previously observed in the 16S rRNA phylogeny, He02^T^ strain clustered with *H. floricola* DSM 23037^T^, but the phylogenetic distance between the two genomes suggested that He02^T^ constitute a different species (Fig. 2). In order to ascertain the taxonomic status of strain He02^T^, average nucleotide identity (ANI) values were calculated with FastANI [23] as implemented in Proksee [24] and OrthoANI [25]. The ANI values between strains He02^T^ and *H. floricola* DSM 23037^T^ were 87.44 and 86.02, with FastANI and OrthoANI, respectively. These values are below the proposed threshold (95–96%) for distinguishing between bacterial species [23,26]. In addition, digital DNA–DNA hybridization (dDDH) was carried out with Type Strain Genome Server [27]. A value of 30.5% (confidence interval 28.2–33.1%) was obtained with formula *d*_4_, which is recommended for incomplete genomes [28]. This value lies also below the threshold for bacterial species.

## Functional characterization of *H. saturejae* genome

The analysis of the He02^T^ genome mobilome was carried out using Islandviewer 4 [29] for genomic islands, ISfinder [30] (http://www-is.biotoul.fr) for IS elements and PHASTEST [31] for prophages. The analysis of the metabolic capabilities of *H. saturejae* He02^T^ was carried out using BlastKOALA [32]. A search for carbohydrate-active enzymes was performed with dbCAN3 [33]. The presence of a CRISPR anti-phage defence system was examined with CRISPRCasFinder [34], and the search for antimicrobial resistance systems was carried out using ResFinder v2.0 [35]. Additionally, analyses of antibiotic resistance models and bioactive secondary metabolite gene clusters were conducted using the ARTS 2.0 [36] program and antiSMASH 7.0 [37].

The searches for genomic islands or IS elements did not detect any genetic mobile element. The sequence was also inspected for putative plasmid replication proteins. Five putative plasmid replication proteins were found in four contigs (Table S1, available in the online Supplementary Material), suggesting the presence of plasmids in this strain corresponding to contigs 3, 4, 5 and 6. However, this point remains hypothetical until the current assembly is verified. The analysis with PHASTEST detected two phage regions (Fig. S1; contig 4; acc. NZ_JAWMWG010000003.1; R4Y45_06025 to R4Y45_06270, and contig 7; acc. NZ_JAWMWG010000006.1; R4Y45_07195 to R4Y45_07295). The analysis of the gene content indicates that the first region encompasses modules for replication, assembly, capsid and lysis. However, genes for integration and immunity were not detected. The second region only contained genes encoding for capsid components and lysis. Therefore, these regions may constitute remnants of prophages no longer functional. The search for a CRISPR anti-phage system failed to detect any putative CRISPR cluster in *H. saturejae* He02^T^. In contrast, *H. floricola* DSM 23037^T^ encodes a CRISPR/Cas9 cluster (genes FC86_GL000508 to FC86_GL000511). Subsquent tblastn searches using the *H. floricola* genes against the genome of strain He02^T^ confirmed the absence of a CRISPR cluster in this strain.

The analysis with BlastKOALA mapped 58% of the annotated genes and suggested that strain He02^T^ had limited metabolic capabilities (Fig. S2). As examples, carbohydrate metabolism accounted for 7% of mapped genes, and amino acid metabolism accounted for 5%.

## Carbohydrate metabolism

Strain He02^T^ encodes the genes required for the Embden-Meyerhof-Parnas homofermentative pathway (Fig. S3). Regarding the metabolism of pentoses, strain He02^T^ encodes enzymes required for the conversion of glucose or fructose into pentoses (Fig. S4) but lacks the enzymes required for the Entner-Doudoroff pathway, transaldolase (EC 2.2.1.2) and transketolase (EC 2.2.1.1) which agrees with its inability to grow on pentoses as carbon sources (see below). Notwithstanding, He02^T^ encodes a putative phosphoketolase (EC 4.1.2.9), which splits d-xylulose 5-phosphate into glyceraldehyde 3-phosphate and acetyl-phosphate (R4Y45_01830, not mapped in BlastKOALA). Whether this enzyme is actually expressed remains to be determined. Carbohydrate biosynthetic capabilities are also limited as strain He02^T^ lacks a gluconeogenic pathway (Fig. S3).

The production of d- and l-lactic was measured in MRS by using Enzytec™ Liquid D-/L-Lactic acid kit (Biopharm AG, Darmstadt, Germany) according to the manufacturer's instructions. Briefly, the supernatant of an overnight culture was obtained by centrifugation and filtered through a 0.22 µm pore-size filter. The filtered supernatant was appropriately diluted and used to determine the presence of l-lactic acid or d-lactic acid by measuring the increase in NADH resulting from reactions catalyzed by either d-lactate dehydrogenase or l-lactate dehydrogenase. The results revealed that He02^T^ produced L-lactic acid from glucose with no detectable levels of d-lactic acid (Table 1). This finding agrees with the previous observation that *H. floricola* only produces l-lactic acid from glucose [5]. However, strain He02^T^ encodes two putative proteins (R4Y45_02115 and R4Y45_05745) with significant similarity to putative d- lactate dehydrogenases. No homologs are found in the genome of *H. floricola* DSM 23037^T^. Both proteins belong to the D-2-hydroxyacid NAD(+)-dependent dehydrogenase family, which encompasses various enzymes acting on different D-hydroxy-carboxylic acids. Consequently, amino acid sequence alone is insufficient to predict their enzymatic activities.

**Table 1.** Growth characteristics of *H. saturejae* He02^T^

Condition	Specific growth rate (h^−1^)	Maximal OD	Lactic acid isomer	MIC (µg ml^−1^)
Growth in MRS			L	
Growth in MRS (pH 6.5)	0.041±0.002	0.303±0.028		
Growth in MRS (pH 5.5)	0.002±0.001	0.060±0.018		
Growth in MRS (pH 4.5)	–	–		
Growth in MRS (pH 6.5; 0.5% NaCl)	0.070±0.007	0.411±0.059		
Growth in MRS (pH 6.5; 1% NaCl)	0.033±0.004	0.275±0.04		
Growth in MRS (pH 6.5; 2% NaCl)	–	–		
Growth in FYP (pH 6.5; aerobic)	0.003±0.001	0.062±0.012		
Growth in FYP (pH 6.5; anaerobic)	0.167±0.046	0.350±0.011		
Growth in GYP (pH 6.5; aerobic)	0.071±0.004	0.434±0.022		
Growth in GYP (pH 6.5; anaerobic)	0.236±0.011	1.193±0.033		
Growth in GYP (pH 6.5; anaerobic; pyruvate 0.25%)	0.234±0.011	1.182±0.032		
Growth in GYP (pH 6.5; anaerobic; pyruvate 0.5%)	0.234±0.009	1.168±0.022		
Ampicillin				0.25
Clindamycin				0.016
Chloramphenicol				2
Erythromycin				0.25
Gentamycin				128
Kanamycin				>256
Tetracycline				1
Vancomycin				0.38

-),(-−), nNo growth.

The limited capacity of the utilization of carbohydrates is also suggested by the apparently limited range of sugar transporters encoded by this strain. Strain He02^T^ carries an incomplete and thus non-functional, phospho*enol*pyruvate-dependent sugar phosphotransferase system (PTS), an important bacterial sugar transport system involved in the transport of a multitude of substrates [38]. He02^T^ showed the absence of a *ptsI* gene in its genome, encoding the phosphocarrier protein enzyme I, while it had a *ptsH* gene (locus R4Y45_05695), encoding the histidine-containing phosphocarrier protein HPr of the PTS. It also encoded a few IIA (locus R4Y45_02965) and IIC (loci R4Y45_00085, R4Y45_00770, R4Y45_00775, R4Y45_05800 and R4Y45_06540) sugar-specific components of PTS transporters, although they appear incomplete or as remnants and did not seem to conform functional PTSs (Fig. S5). The inspection of the genome of *H. floricola* DSM 23037^T^ showed that these characteristics (e.g. the absence of *ptsI* but the presence of *ptsH* gene from the PTS) were also shared with that species, which has also been shown to only utilize glucose and fructose from a battery of 14 sugars tested [5]. The analysis of ABC transporters also evidenced the absence of characterized carbohydrate ABC transporters (Fig. S6). According to this, the search for carbohydrate-active enzymes present in the He02^T^ genome using dbCAN3 [33] only revealed the presence of three putative enzymes showing glycosylhydrolase domains from the GH73 and GH32 families: genes R4Y45_02055 and R4Y45_05890. Both enzymes contain signal peptides as predicted by SignalP-6.0 [39], indicating that they are possibly extracellular enzymes involved in cell wall turnover (endo-*β*-*N*-acetylglucosaminidases). In addition, strain He02^T^ encodes a putative invertase (R4Y45_02190). This enzyme lacks a signal peptide as predicted by SignalP-6.0, suggesting that it is an intracellular enzyme. The apparent absence of sucrose transporters would account for the inability of He02^T^ to grow on sucrose.

The analysis of pyruvate catabolic pathways showed that strain He02^T^ lacks alternative pathways to lactate dehydrogenase such as pyruvate dehydrogenase, pyruvate:formate lyase or pyruvate oxidase (Fig. S7). Of note, as many other LAB, strain He02^T^ encodes a putative malolactic enzyme (R4Y45_05065) that would let it use malic acid as a source of metabolic energy [40]. Strain He02^T^ also encode putative phosphate acetyltransferase and acetate kinase (R4Y45_03230 and R4Y45_03335, respectively) that would let it obtain ATP from acetyl-CoA.

## Amino acid metabolism

The analysis of the genome suggests that *H. saturejae* He02^T^ requires a number of amino acids. Strain He02^T^ encodes putative ABC transporters for Arg, Asp, Cystine, Gln, Glu, His and Lys in addition to an Opp oligopeptide ABC transporter (Fig. S6). The inspection of the metabolic pathways suggests that strain He02^T^ can interconvert Asp and Asn and Glu and Gln, respectively (Fig. S8), and can synthesize Gly, Ser and Thr from Asp (Fig. S9). Furthermore, strain He02^T^ encodes the genes required to synthesize Met from Asp, providing that a source of sulphur such as thiosulfate or hydrogen sulphide is available, whereas it cannot possibly synthesize Cys (Fig. S10). The pathway for Lys biosynthesis from Asp is also present (Fig. S11). In contrast, biosynthetic pathways for Ala, Arg, aromatic amino acids (Phe, Trp and Tyr), branched-chain amino acids (Leu, Ile and Val) and Pro are incomplete or absent.

The presence of oligopeptide transporters suggests that strain He02^T^ may obtain essential amino acids from internalized peptides that are subsequently hydrolysed by intracellular peptidases. In fact, strain He02^T^ encodes a varied array of aminopeptidases and endopeptidases (Table S2). In contrast, strain He02^T^ does not encode any cell envelope proteinases (CEPs), which are a key component of the proteolytic system in other LAB [41]. CEPs break extracellular proteins into peptides that are subsequently uptaken and degraded by intracellular peptidases. The absence of these proteinases suggests that *H. saturejae* relies on the proteolytic activities of other organisms present in its habitat.

## Secondary metabolism

Analysis of secondary metabolism identified a remnant of a putative bacteriocin cluster consisting of an *N*-terminal truncated sensor histidine kinase (CitA, COG3290; R4Y45_RS01320), a LytR/AlgR family response regulator (R4Y45_RS01325; COG3279) and C-t terminal putative peptidase-containing ABC-type bacteriocin transporter (R4Y45_RS01330; SunT, COG2274). This cluster is also present in *H. floricola*, although both the histidine kinase and the ABC transporter are apparently intact in this genome. Comparison with other LAB-encoding homologous genes indicate that key genes such as those encoding bacteriocin-like peptides and immunity proteins are missing in strain He02^T^ (Fig. S12) and *H. floricola*.

The analysis also identified two genes putatively involved in terpene metabolism: a putative phytoene synthase (R4Y45_02165; COG1562) and a putative phytoene desaturase (R4Y45_02170; COG1233). Since strain He02^T^ possesses a complete terpenoid backbone biosynthesis pathway (Fig. S13), these genes might produce a carotenoid pigment from farnesyl diphosphate that would account for the yellow pigmentation of He02^T^ cells (see below).

No antibiotic-producing pathways or mobilizable antibiotic resistance genes were detected in the genome of strain He02^T^. Resistance to antibiotics was also assayed by using ETEST^®^ strips (bioMérieux) deposited onto MRS agar plates overlayed with MRS soft agar (0.8% w/v) containing He02^T^ cells at an OD_595_ of 0.2. The plates were incubated at 30 °C for 24 h. The strips provide a predefined antibiotic gradient that is used to determine the MIC. Strain He02^T^ was resistant to gentamycin and kanamycin, whereas it was sensitive to ampicillin, clindamycin, chloramphenicol, erythromycin, tetracycline and vancomycin (Table 1). Intrinsic resistance to aminoglycosides, including gentamycin and kanamycin, is common in *Lactobacillaceae* [42,43], which may account for the resistance observed in the absence of specific resistance genes.

On the other hand, two putative multidrug ABC exporter-encoding genes (R4Y45_00495 and R4Y45_00500; MdlB superfamily, COG1132) were identified. In addition, a putative antimicrobial resistance gene cluster belonging to the Bce-like family was identified. Bce-like systems, named after the BceRSAB system from *Bacillus subtilis*, are involved in resistance against a number of antimicrobial peptides [44,45]. They typically consist of a two-component regulatory system and a cognate ABC transporter. In strain He02^T^, genes R4Y45_05675, R4Y45_05680 and R4Y45_05685 encode a putative ABC ATPase subunit, a response regulator and a sensor histidine kinase, respectively. Gene R4Y45_05675 encode a hypothetical membrane protein. blast searches against *Lacticaseibacillus paracasei* BL23, which encode two BceRSAB systems (ApsRSAB and PsdRSAB) and an additional Bce-like ABC transporter (DerAB) previously characterized [46,47], revealed that proteins with highest scores corresponded to the Bce proteins encoded by this organism (Table S3). The best scores for genes R4Y45_05675, R4Y45_05680 and R4Y45_05685 were registered with their Aps counterparts. Furthermore, both clusters shared the same structure (Fig. S14). In contrast, no significant hit in *Lc. paracasei* BL23 was found for gene R4Y45_05670. No conserved motif is recognized in this protein. However, the analysis con TOPCONS [48] predicts that the protein contains ten transmembrane helices and BlastP searches find significant hits with proteins annotated as ABC permeases such as YybP from *Amyloactobacillus* (WP_056945950; 100% coverage, 21.95% identical residues) and LolE from *Sutcliffiella halmapala* (WP_169864955; 99% coverage; 22.65% identical residues). These results suggest that R4Y45_05670 might constitute a functional ABC transporter together with R4Y45_05675.

In summary, the analysis of the genome sequence indicates that *H. saturejae* He02^T^ has limited metabolic capabilities, relying mostly on homofermentative fermentation of monosaccharides as an energy source. Like other LAB, *H. saturejae* requires a number of amino acids that may possibly be obtained mainly from the hydrolysis of uptaken extracellular peptides. Of note, its capacity to produce a carotenoid pigment may be a photoprotective mechanism against sunlight exposure in flowers.

## Growth characteristics of *H. saturejae*

**Fig. 3.**
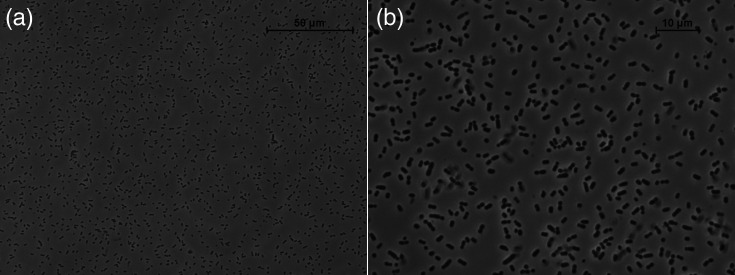
Microscopy images of *H. saturejae* He02^T^ cultured in MRS medium. (**a**) 40× magnification. (**b**) 100× magnification.

When cultivated in MRS (BD Difco), a general medium for LAB, *H. saturejae* He02^T^ grew as non-motile short rods (1.67±0.32 µm×0.92±0.11 µm), typically occurring in pairs (Fig. 3). Pelleted cells are pale yellow although this characteristic is not perceptible in colonies due to their small size. Growth in liquid MRS (pH 6.5) at 30 °C was slow (growth rate of 0.041±0.002 h^−1^ and a final OD_600nm_ of 0.30±0.03 after 24 h) and sharply decreased when the pH of the medium was adjusted to 5.5 (growth rate of 0.002±0.001 h^−1^), with no growth occurring at pH 4.5 (Table 1). The presence of 0.5% NaCl (w/v) enhanced growth compared to MRS alone (growth rate of 0.070±0.007 h^−1^ and final OD_600nm_ of 0.41±0.06), but growth was reduced at 1% NaCl (w/v) and absent at 2% NaCl (w/v). Growth was observed at 15 °C after 48 h of incubation, but not at 42 °C. Strain He02^T^ displayed a homofermentative metabolism, and despite being isolated in FYP medium, designed for the isolation of fructophilic LAB [8], it did not show a fructophilic behaviour, and it grew faster when the carbon source of this medium (fructose) was replaced by glucose (GYP medium; Table 1). Strain He02^T^ grew poorly under aerobic (air) conditions, whereas limiting the oxygen in the liquid growth medium by the addition of Oxyrase (Merck) enhanced growth (Table 1). Supplementation of the growth medium with pyruvate did not affect growth parameters (Table 1).

Strain He02^T^ possessed a very limited range of the utilization of carbohydrate substrates and only glucose and fructose tested positive from the 49 carbohydrates present in API50 CH galleries. The narrow range of utilized carbohydrates appears to be a characteristic of the *Holzapfeliella* genus and probably reflects the adaptation of these bacteria and other niche-related LAB, such as fructophilic LAB [3], to proliferate in very specialized niches like the nectar of flowers, which are rich in fermentable sugars but possess a limited variety of them.

Based on the phylogenetic analyses, ANI and dDDH low values compared to the closest species, *H. floricola*, we conclude that strain He02^T^ constitute a new species, for which the name *Holzapfeliella saturejae* sp. nov. is proposed. The type strain is He02^T^ (=CECT 31001^T^=DSM 117324^T^=CCM 9395^T^), isolated from flowers of *S. montana* L.

### Description of *Holzapfeliella saturejae* sp. nov.

*Holzapfeliella saturejae* (sa.tu.re’jae. N.L. fem. n. *saturejae*, named after the plant genus *Satureja*).

Cells are homofermentative, Gram-stain-positive, catalase-negative, non-motile, short rod-shaped and typically occur in pairs. They grow in MRS broth in anaerobic, micro-aerobic or aerobic conditions, although growth is stimulated in anaerobiosis. It can grow at 15 °C but not at 42 °C. It grows poorly at pH 5.5 and not at pH 4.5. It tolerates NaCl concentration in MRS broth up to 1%, with optimal growth attained with 0.5% NaCl. On MRS agar plates, *H. saturejae* formed round and smooth colonies that were translucent and less than 1 mm in diameter after 2 days of growth at 30 °C. *H. saturejae* only fermented glucose and fructose. Using the API50 CH test kit, it cannot ferment glycerol, erythritol, d-arabinose, l-arabinose, d-ribose, d-xylose, l-xylose, d-adonitol, methyl *β*-d-xylopyranoside, d-galactose, d-mannose, l-sorbose, l-rhamnose, dulcitol, inositol, d-mannitol, d-sorbitol, methyl d-mannopyranoside, methyl *α*-d-glucopyranoside, *N*-acetyl-glucosamine, amygdalin, arbutin, aesculin, salicin, d-cellobiose, d-maltose, d-lactose, d-melibiose, d-saccharose, d-trehalose, inulin, d-melezitose, d-raffinose, starch, glycogen, xylitol, gentiobiose, d-turanose, d-lyxose, d-fucose, l-fucose, d-arabitol, l-arabitol, gluconate, 2-keto-gluconate or 5-keto-gluconate.

The type strain He02^T^ (=CECT 31001^T^=DSM 117324^T^=CCM 9395^T^) was isolated from *S. montana* L. (*Lamiaceae*) flowers. It has a G+C content of 34.99 mol%. The GenBank accession number for the 16S rRNA gene and the genome assembly of He02^T^ are OR978308 and JAWMWG000000000, respectively.

## supplementary material

10.1099/ijsem.0.006654 Uncited Supplementary Material 1.

## Abbreviations

ANI: average nucleotide identity
CEPs: cell envelope proteinases
dDDH: digital DNA–DNA hybridization
LAB: lactic acid bacteria
MRS: man, rogosa and sharpe
NCBI: National Center for Biotechnology Information
PTS: phospho*enol*pyruvate-dependent sugar phosphotransferase system
