# Supplementary material for: Holzapfeliella saturejae sp. nov. isolated from flowers of winter savoury Satureja montana L
Source: Int J Syst Evol Microbiol. 2025 Jan 22;75(1):006654. doi: 10.1099/ijsem.0.006654 (PMC11753529; doi:10.1099/ijsem.0.006654)
Supplement: Uncited Supplementary Material 1. [file ijsem-75-06654-s001.pdf]

Table S1. Putative plasmid DNA replication initiator genes found in *H. saturejae* He02

Accession	Gene tag	Family	Pfam
NZ_JAWMWG010000002	R4Y45_05995	Rep_3	pfam01051
NZ_JAWMWG010000003	R4Y45_06260	Rep_3	pfam01051
NZ_JAWMWG010000003	R4Y45_06270	Rep_3	pfam01051
NZ_JAWMWG010000005	R4Y45_06825	Rep_3	pfam01051
NZ_JAWMWG010000004	R4Y45_06350	Rep_3	pfam01051

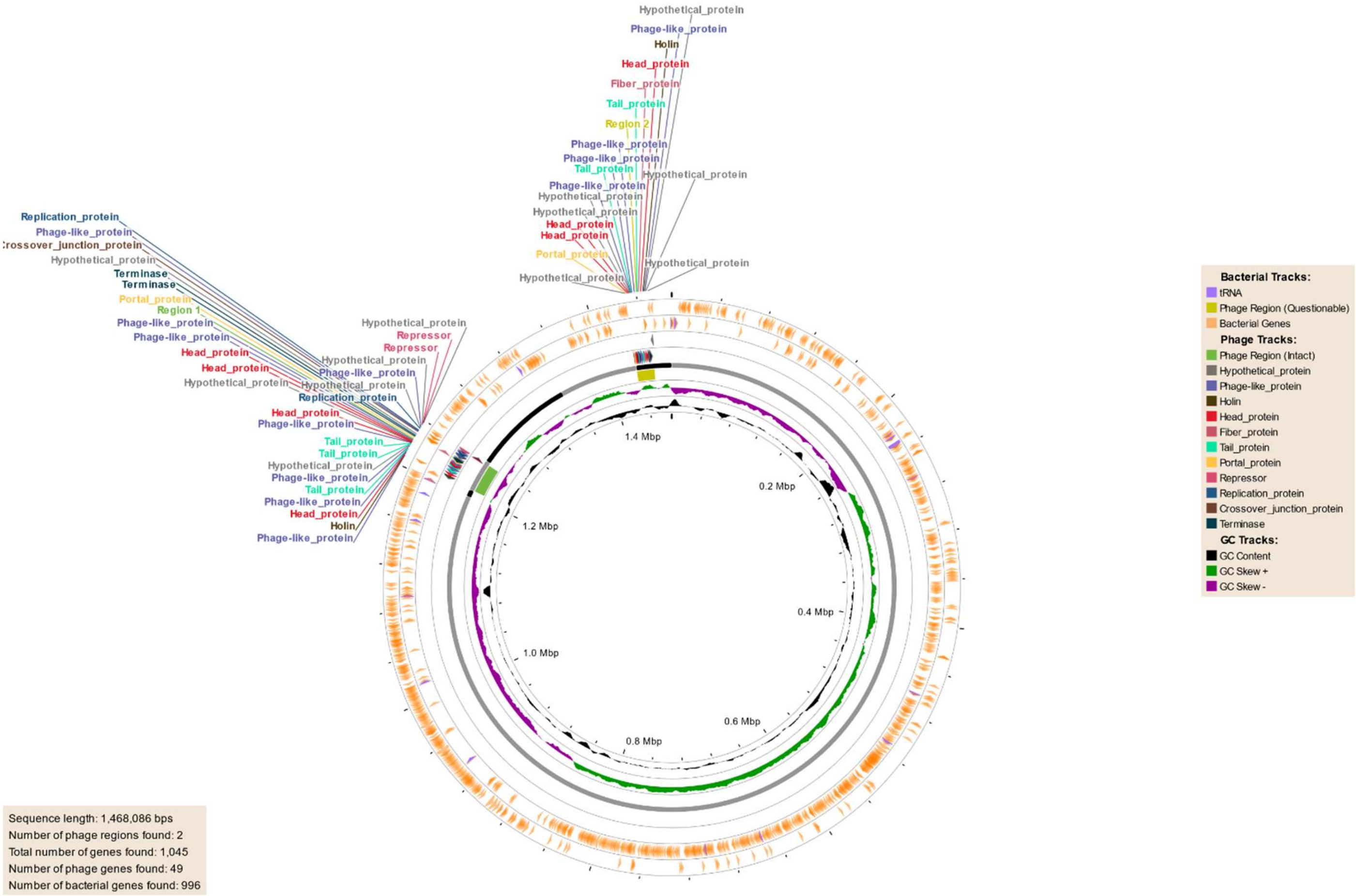

Figure S1. Map of *H. saturejae* genome built with PHASTEST indicating putative phage regions. Assembly of contigs is arbitrary.

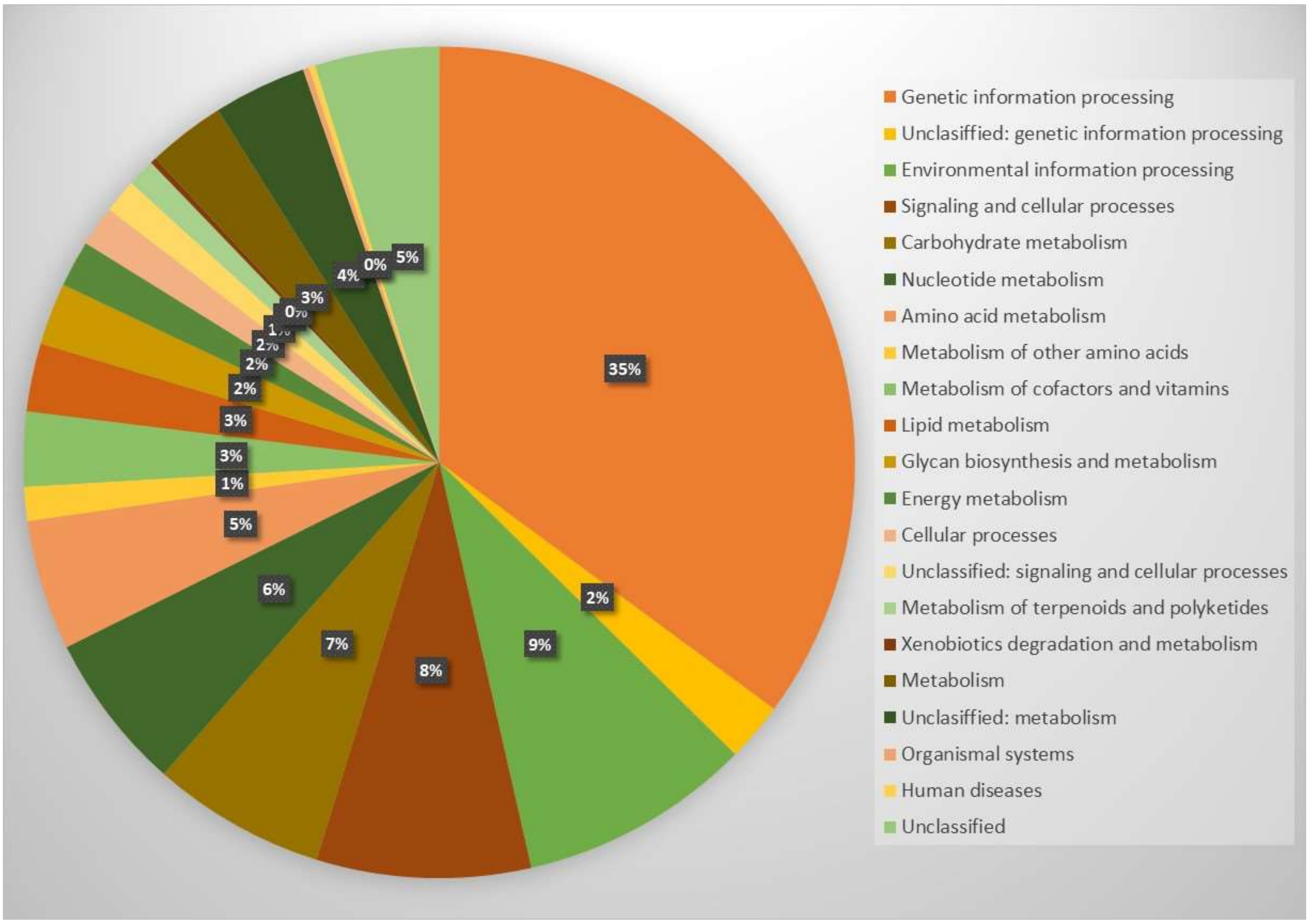

Figure S2. Classification in functional categories of mapped genes of strain He02 with BlastKOALA.

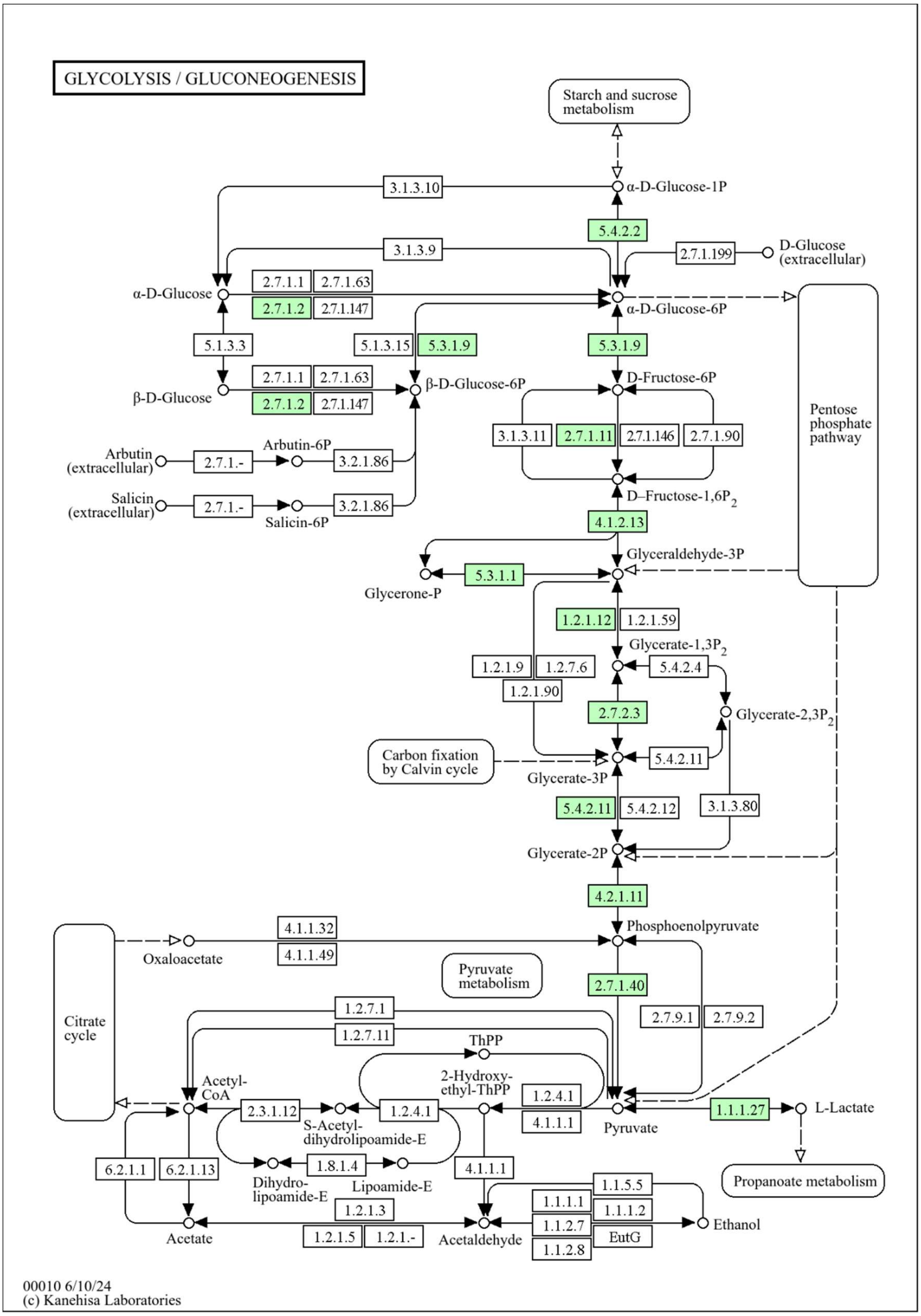

Figure S3. Map of the glycolytic pathway. Enzymes putatively encoded by *H. saturejae* He02 are indicated in green.

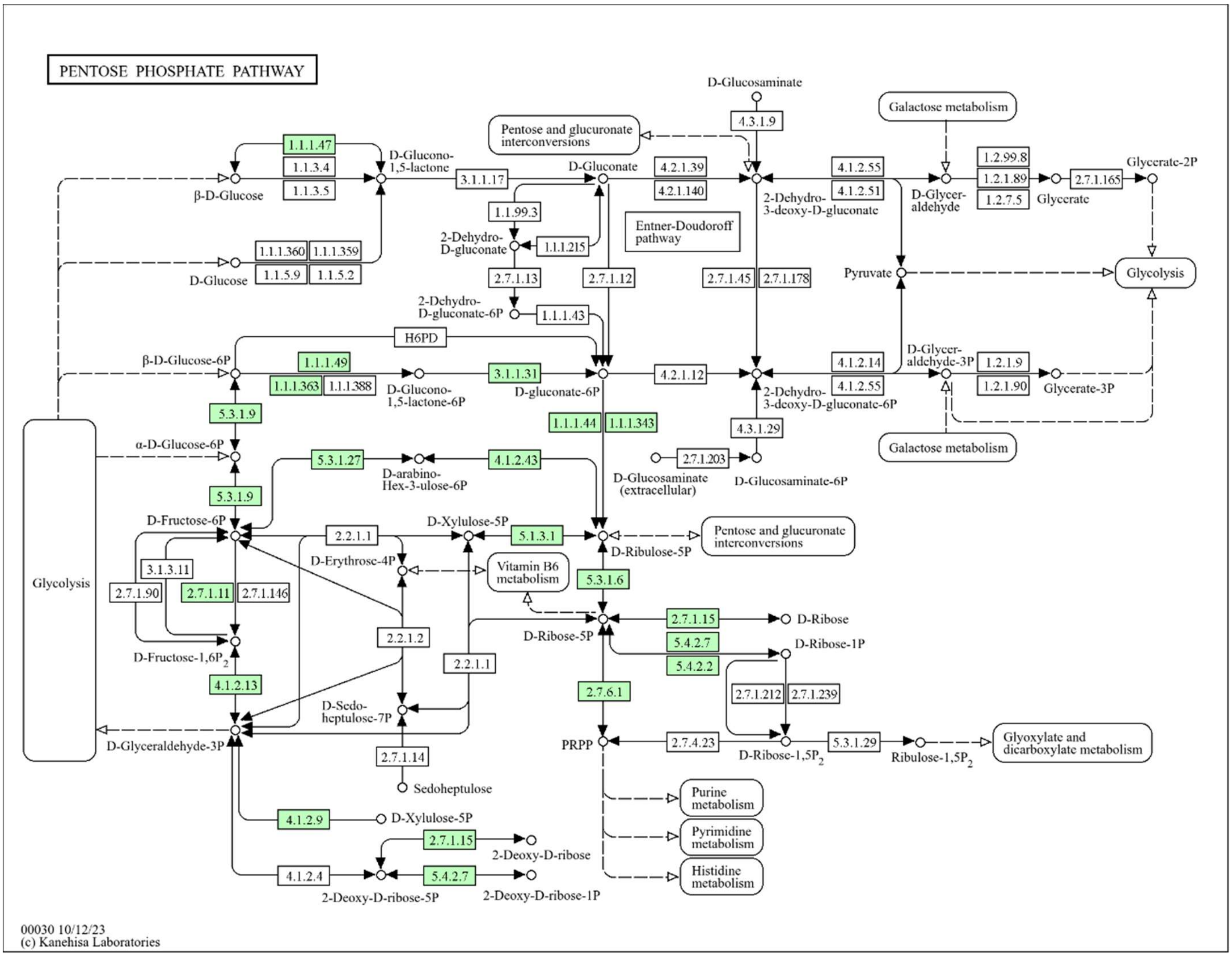

Figure S4. Map of the pentose phosphate pathway. Enzymes putatively encoded by *H. saturejae* He02 are indicated in green.

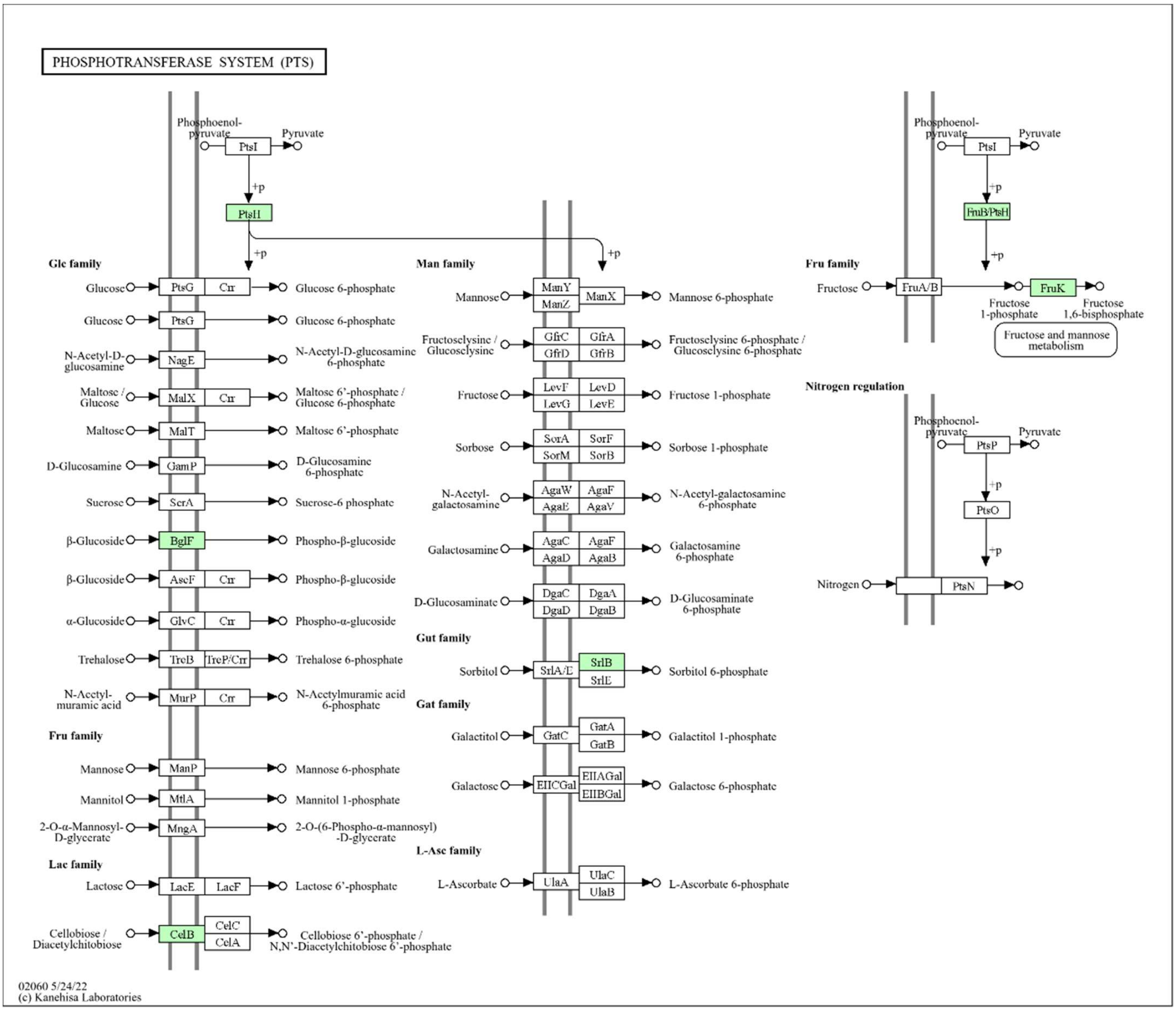

Figure S5. Map of PTS transporters. Enzymes putatively encoded by *H. saturejae* He02 are indicated in green.

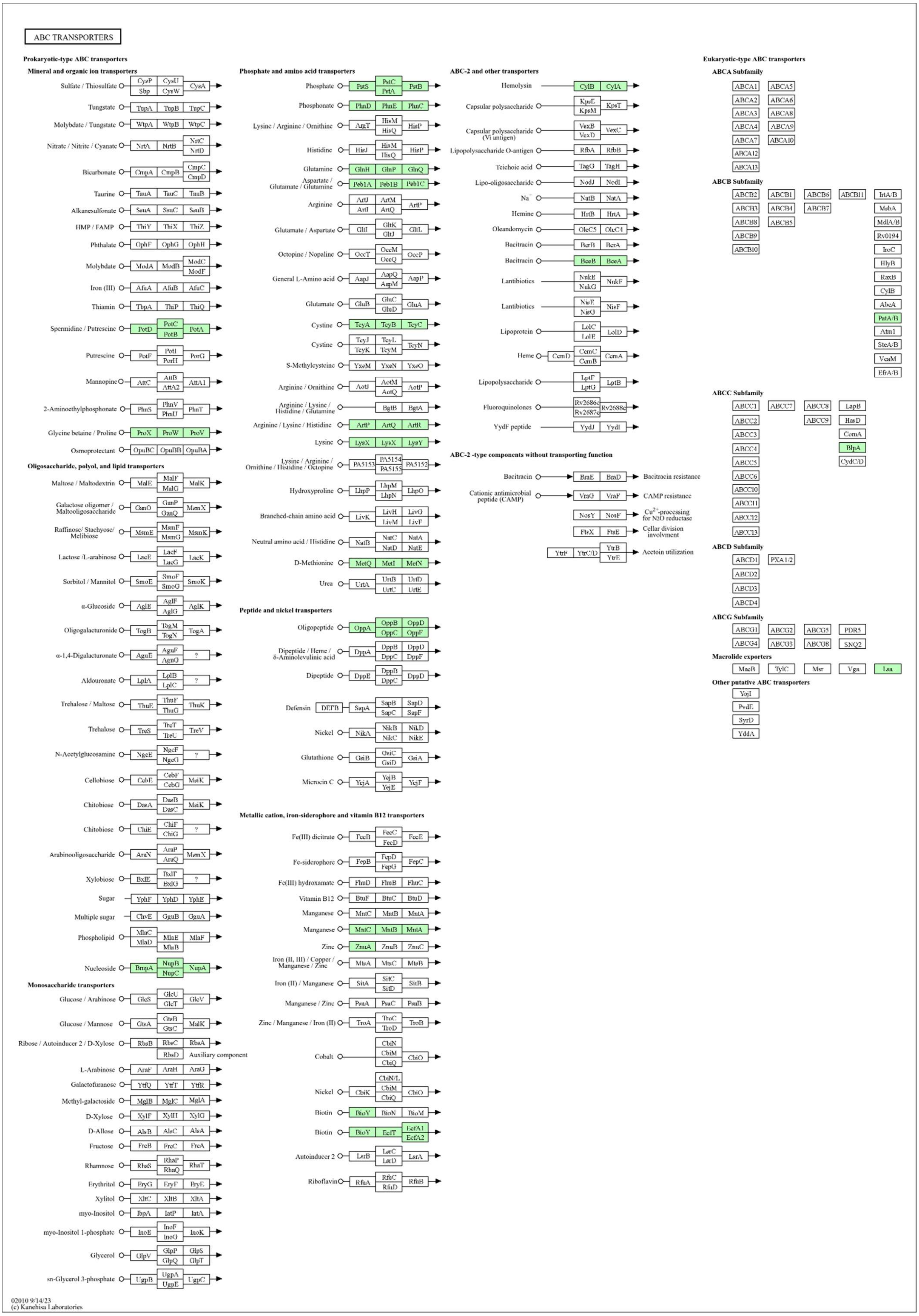

Figure S6. Map of ABC transporters. Enzymes putatively encoded by *H. saturejae* He02 are indicated in green.

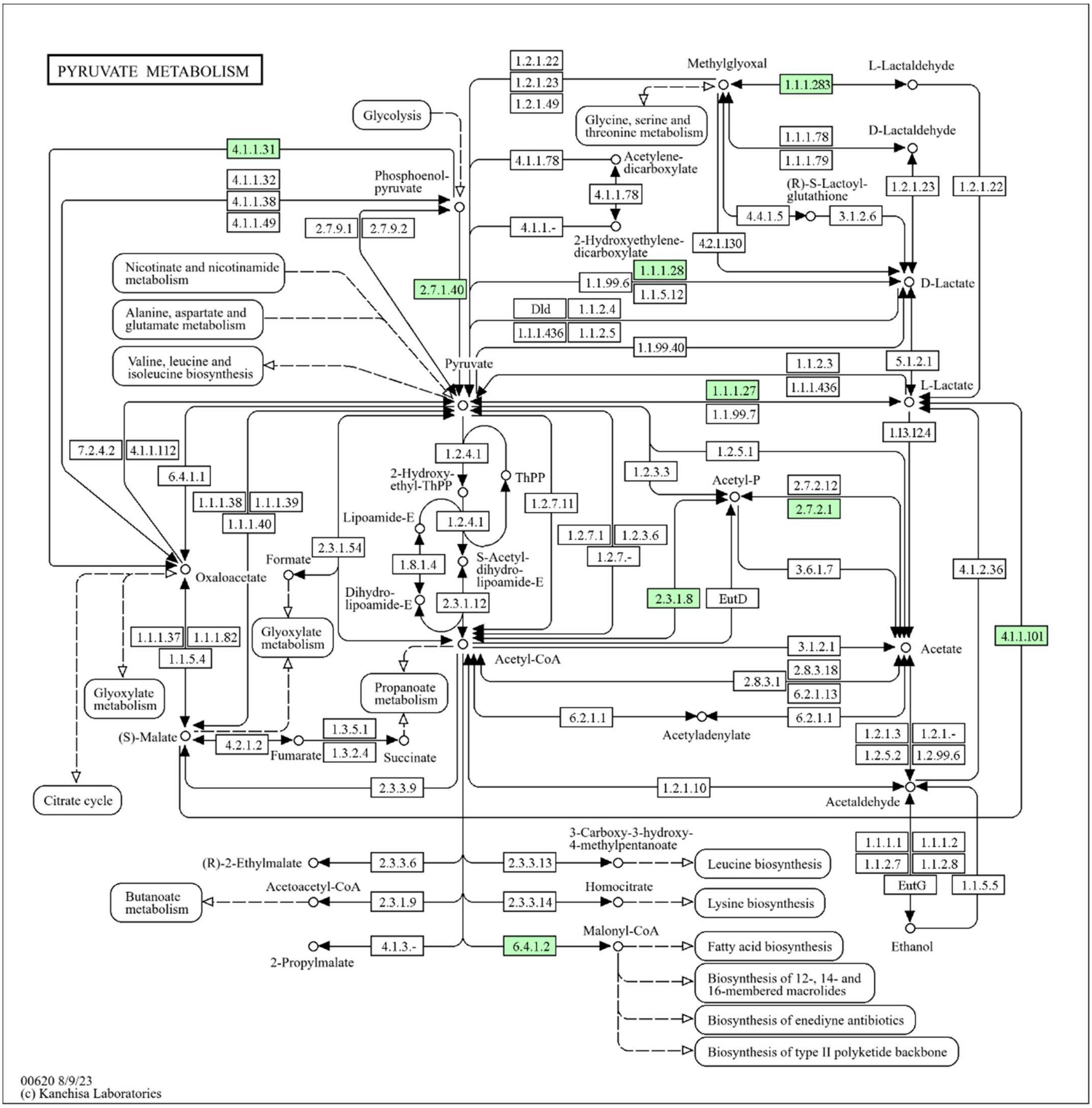

Figure S7. Map of pyruvate catabolic pathways. Enzymes putatively encoded by *H. saturejae* He02 are indicated in green.

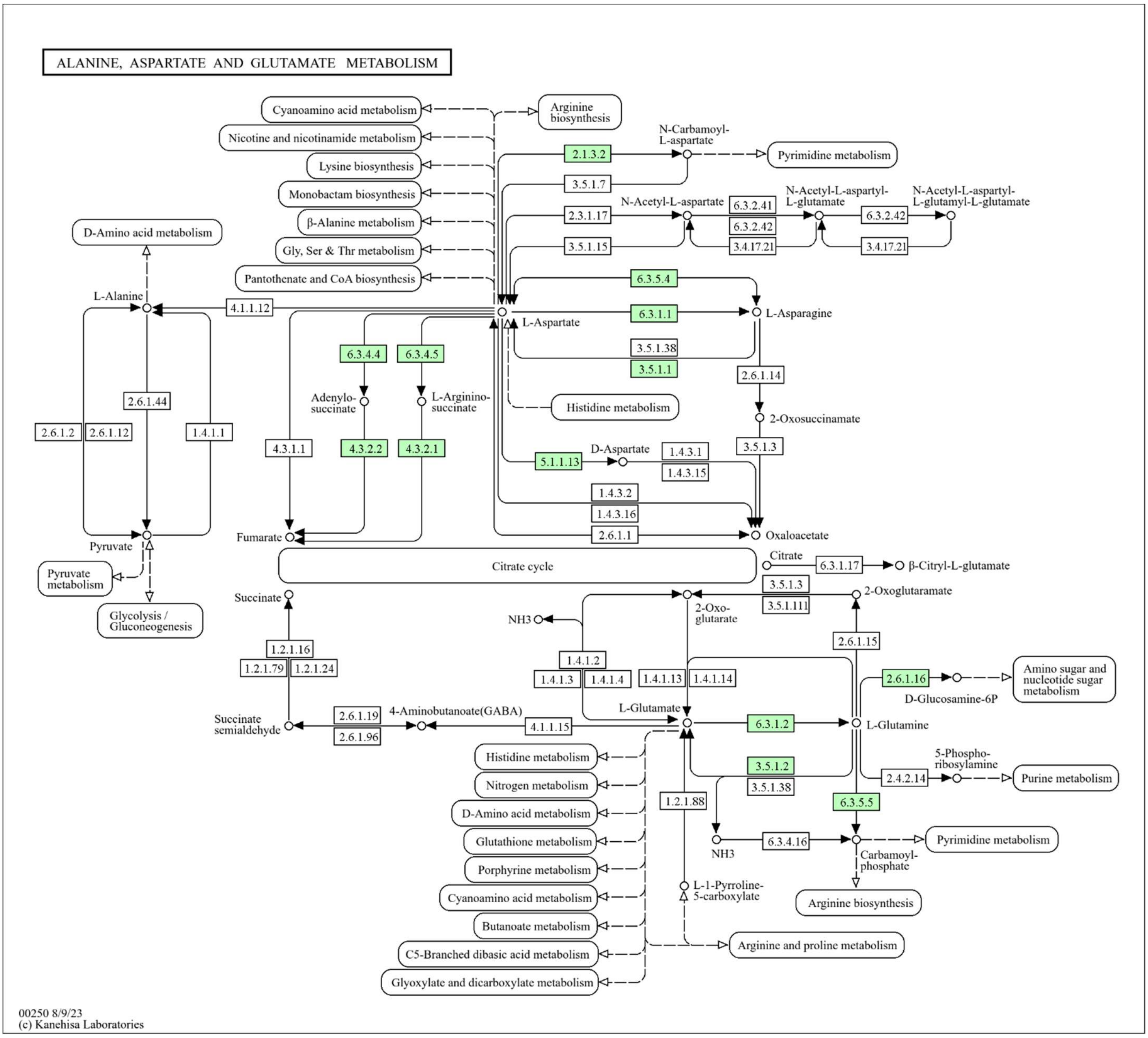

Figure S8. Map of Ala, Asp and Glu metabolic pathways. Enzymes putatively encoded by *H. saturejae* He02 are indicated in green.

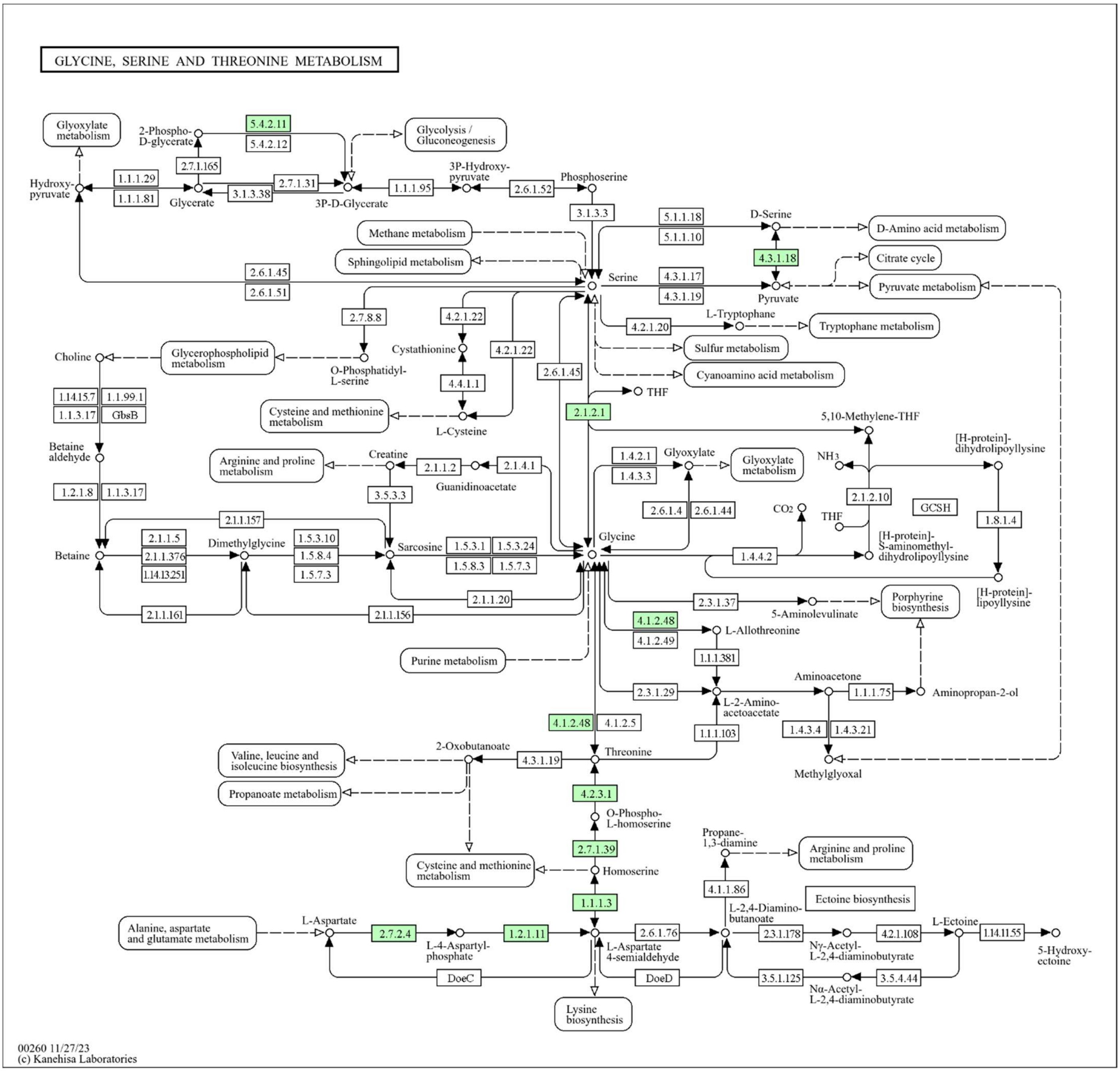

Figure S9. Map of Gly, Ser and Thr metabolic pathways. Enzymes putatively encoded by *H. saturejae* He02 are indicated in green.

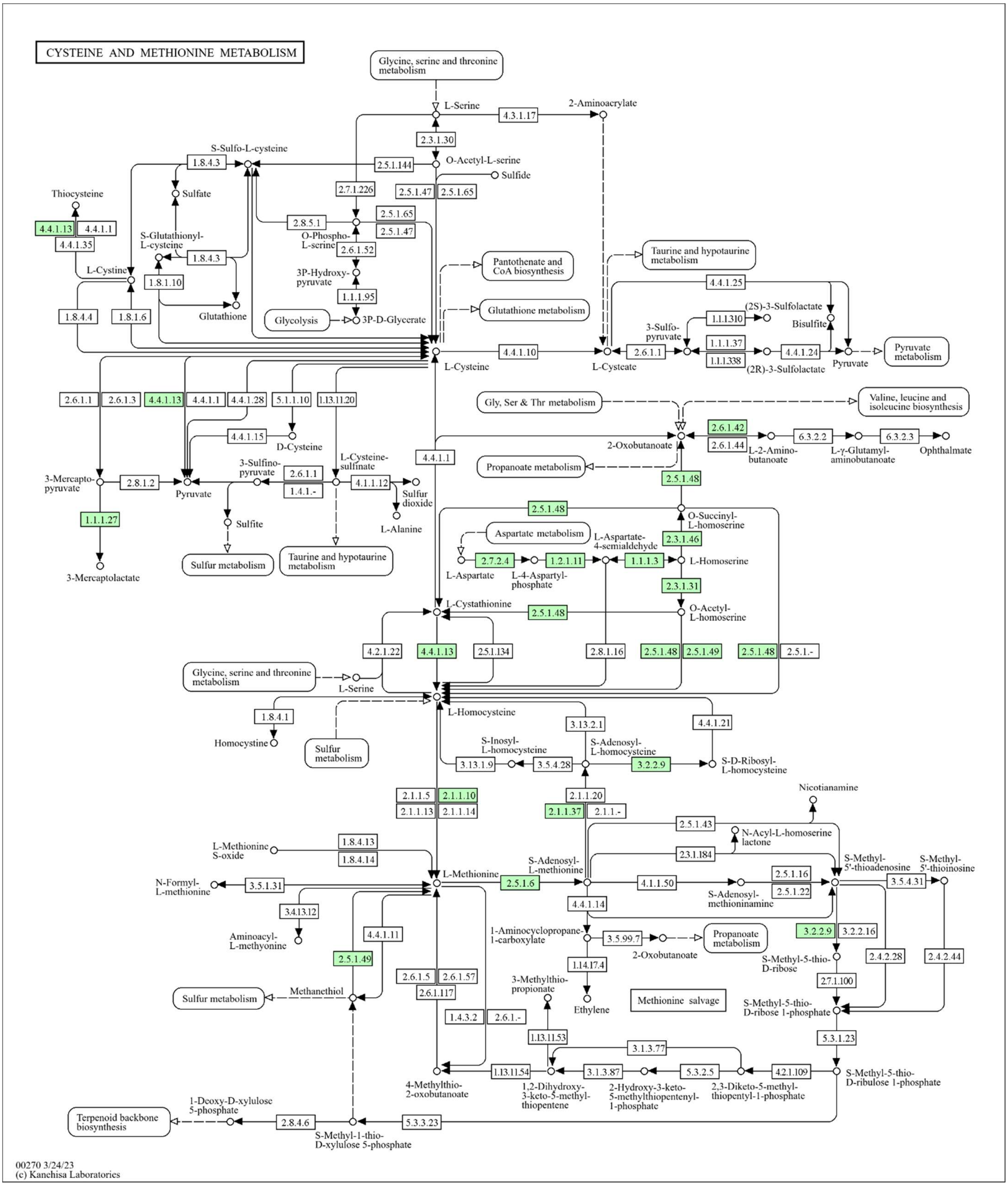

Figure S10. Map of Cys and Met metabolic pathways. Enzymes putatively encoded by *H. saturejae* He02 are indicated in green.

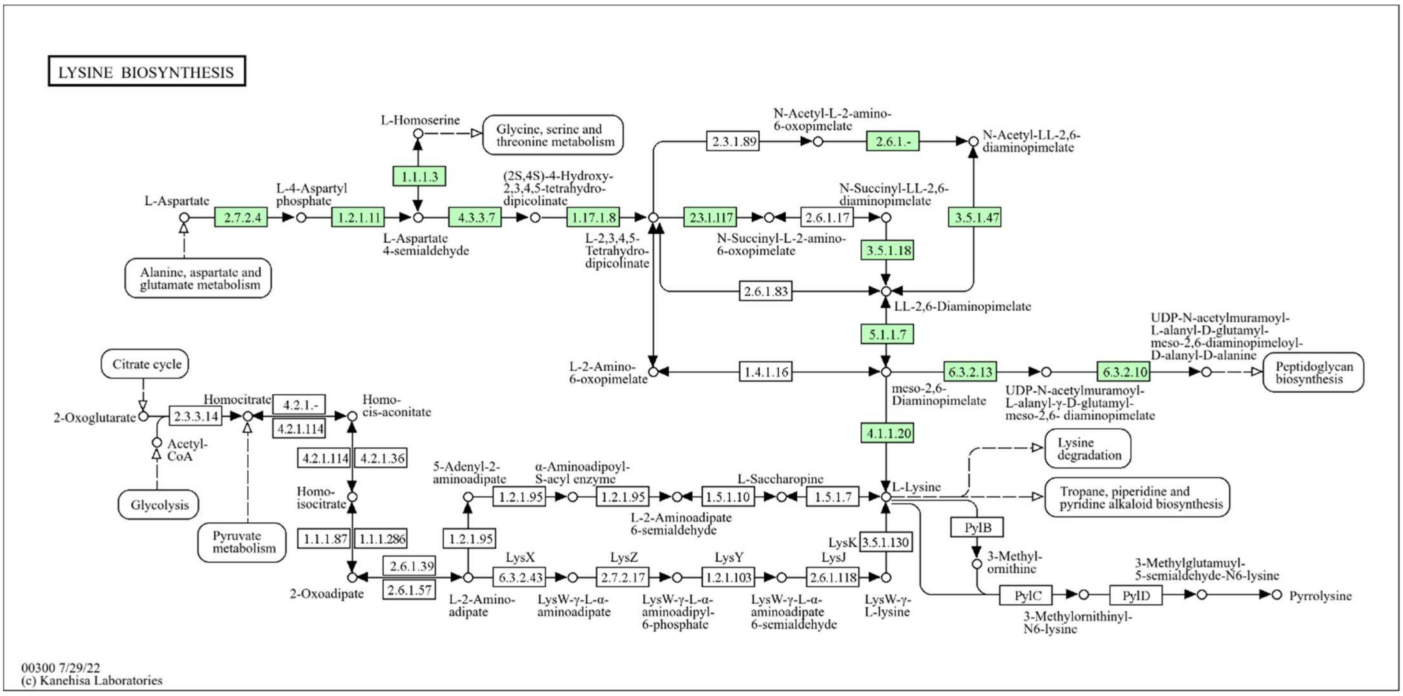

Figure S11. Map of Lys biosynthetic pathways. Enzymes putatively encoded by *H. saturejae* He02 are indicated in green.

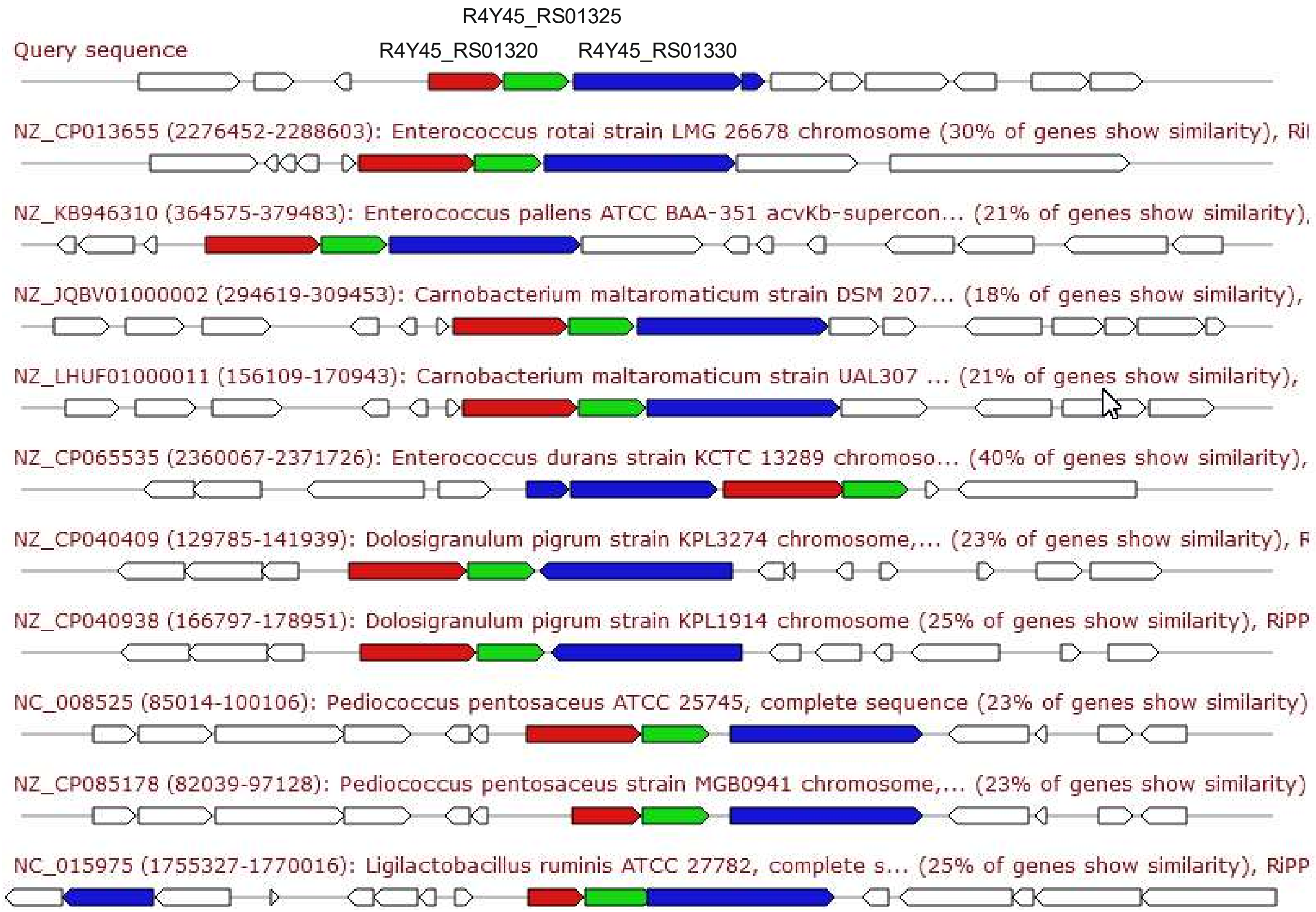

Figure S12. Clusters of homologous genes (indicated by colors) found in strain He02 (Query sequence) and other related lactic acid bacteria using antiSMASH 7.0. Red, sensor histidine kinase; green, response regulator; blue, ABC transporter.

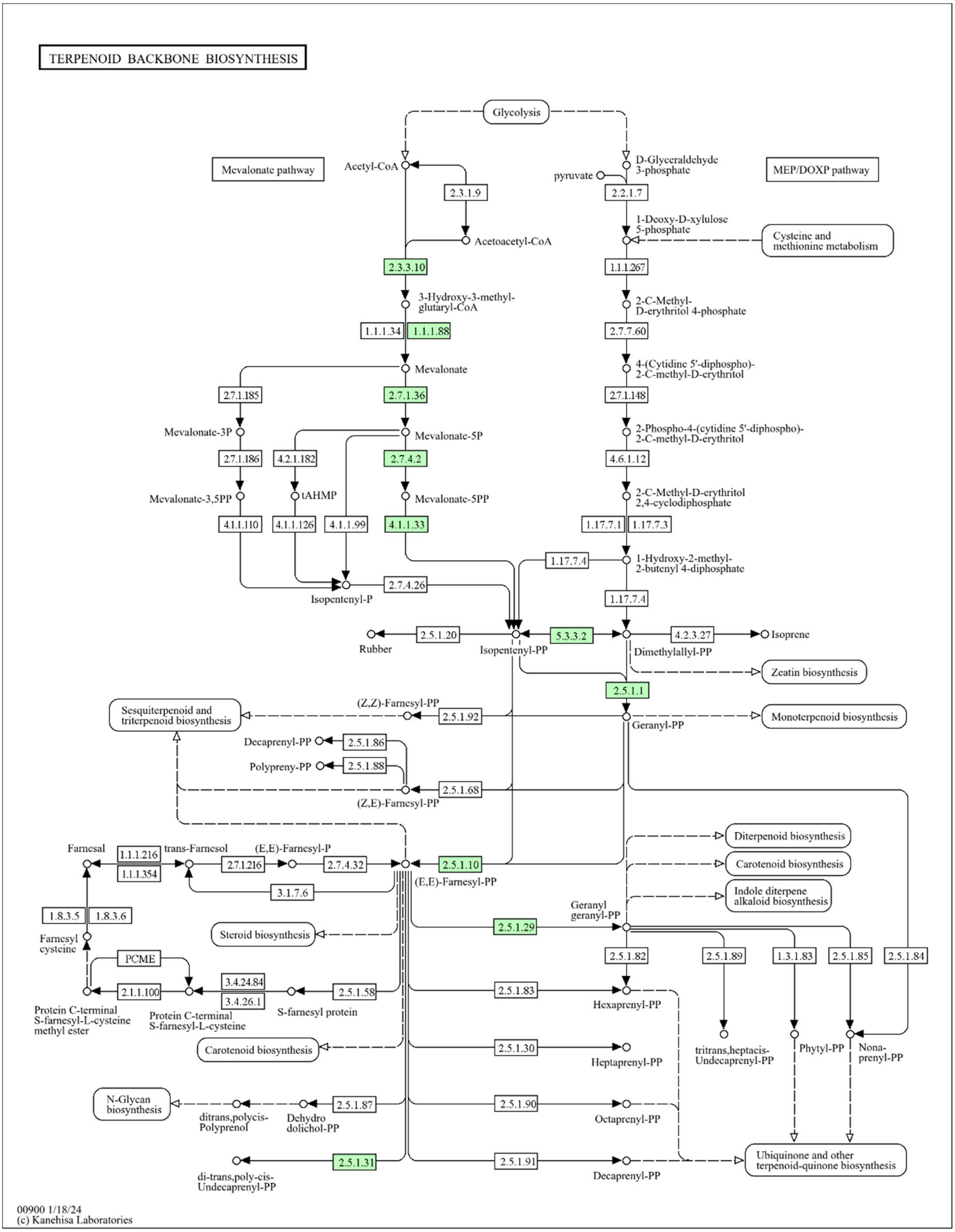

Figure S13. Map of the terpenoid backbone biosynthesis pathway. Enzymes putatively encoded by *H. saturejae* He02 are indicated in green.

Table S2. Predicted peptidases of the proteolytic system of H. saturejae He02*

Gene	Predicted protein	COG	Conserved domain family
R4Y45_05790	Leucyl aminopeptidase	COG2309	
R4Y45_00530	Oligoendopeptidase F	COG1164	cd09608
R4Y45_01270	PepN aminopeptidase	COG0308	cd09601
R4Y45_01655	Oligoendopeptidase F	COG1164	cd09608
R4Y45_01985	PepO endopeptidase	COG3590	cd08662
R4Y45_02660	PepC aminopeptidase	COG3579	cd00585
R4Y45_04510	PepT tripeptide aminopeptidase	COG2195	cd03892
R4Y45_05035	PepP aminopeptidase	COG0006	cd01092
R4Y45_05055	Xaa-Pro dipeptidyl-peptidase	COG2936	
R4Y45_05505	PepV dipeptidase		cd03888
R4Y45_05560	PepP aminopeptidase	COG0006	cd01092
R4Y45_05775	PepM methionyl aminopeptidase	COG0024	cd01086
R4Y45_05790	Leucyl aminopeptidase	COG2309	

* Enzymes putatively involved in cell maintenance and post-translational processing are not included

***Lacticaseibacillus paracasei* BL23 APS**

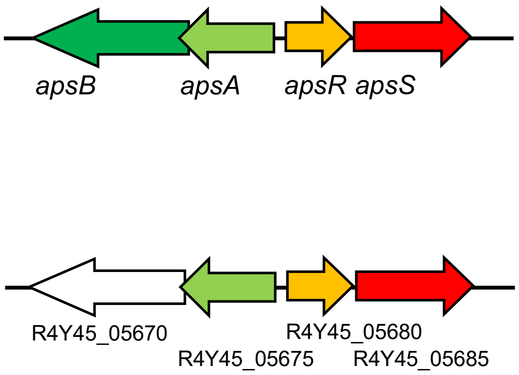

Figure S14. Comparative schematic representation of the APS gene cluster of *Lc. paracasei* BL23 and *H. saturejae* He02. Colors indicate homologous genes.

Table S3. Results of BlastP searches of *H. saturejae* He02 putative Bce system proteins against *Lc. paracasei* BL23 annotated proteins. The three highest scores are shown.

He02 gene	Protein function	*Lc. paracasei* BL23 best hits				
		Gene_tag	Gene	Cover	Identity	E value
R4Y45_05680	Response regulator	LCABL_19600	*apsR*	99%	65.47%	3e-112
		LCABL_16430	*psdR*	97%	40.64%	1e-60
		LCABL_30130	*yycF*	98%	31.58%	5e-38
R4Y45_05685	Histidine kinase	LCABL_19610	*apsS*	96%	42.44%	1e-100
		LCABL_16420	*psdS*	58%	38.16%	2e-39
		LCABL_30120	*hpk3*	60%	26.00%	2e-24
R4Y45_05675	ABC ATPase subunit	LCABL_19590	*apsA*	98%	47.98%	2e-80
		LCABL_16410	*psdA*	98%	43.31%	4e-67
		LCABL_21680	*derA*	95%	44.03%	3e-66
R4Y45_05670	ABC permease subunit	**No significant hit**				
